# Portable devices for the diagnosis of glaucoma: a scoping review

**DOI:** 10.1136/bmjopen-2025-105681

**Published:** 2025-10-21

**Authors:** Farouk Garba, Fatima Kyari, Matthew J Burton, Samuel Harris Adler, Zunaira Khan, Mubarak Bello, Jennifer Evans, Iris Gordon, Victor H Hu, Winifred Nolan

**Affiliations:** 1 London School of Hygiene & Tropical Medicine, London, UK; 2 Department of Ophthalmology, Ahmadu Bello University, Zaria, Nigeria; 3 College of Health Sciences, University of Abuja, Abuja, Nigeria; 4 The University of Edinburgh Medical School, Edinburgh, UK; 5 Faculty of Medicine, Imperial College London, London, UK; 6 Department of Ophthalmology, Ahmadu Bello University Teaching Hospital, Zaria, Nigeria; 7 Moorfields Eye Hospital NHS Foundation Trust, London, UK

**Keywords:** Glaucoma, PUBLIC HEALTH, Health Services Accessibility

## Abstract

**Abstract:**

**Background:**

Glaucoma is a leading cause of irreversible blindness worldwide. Early detection and continuous monitoring are essential to preventing vision loss, yet traditional diagnostic tools remain largely inaccessible in low-resource settings.

**Purpose:**

This scoping review aimed to map the existing evidence on the use of portable devices for the detection, diagnosis and monitoring of glaucoma.

**Methods:**

We conducted a scoping review in accordance with the Joanna Briggs Institute Manual and Preferred Reporting Items for Systematic Reviews and Meta-Analyses extension for Scoping Reviews guidelines. A comprehensive search was performed across major databases to identify studies that evaluated handheld tonometers, portable fundus cameras and visual field testing devices. Data were extracted on study design, population characteristics, devices used, comparators and reported outcomes.

**Results:**

A total of 216 studies published between 1975 and 2024 were included. Most studies (90.3%) were method agreement studies, primarily focused on intraocular pressure (IOP) devices. Only two studies evaluated all three glaucoma assessment domains (IOP, visual fields and fundus imaging). Most studies were conducted in high-income countries, with a smaller number from low- and middle-income countries. Despite variability in performance, many devices demonstrated acceptable agreement with gold standard methods and were assessed in a range of clinical and community settings.

**Conclusion:**

This review highlights the range and characteristics of portable glaucoma devices and their potential for enhancing access to diagnosis and monitoring, particularly in underserved settings. However, the predominance of method agreement studies and the limited integration of multimodal assessments point to gaps in the literature. Future research should focus on comprehensive diagnostic pathways using multiple portable tools and on expanding evaluations in low-resource settings to inform policy and service planning.

**STRENGTHS AND LIMITATIONS OF THIS STUDY**

- The study provides a comprehensive and methodologically rigorous mapping of global evidence on portable devices for glaucoma diagnosis and monitoring.
- Grey literature and trial registries were not systematically searched, which may have resulted in the omission of unpublished or ongoing studies.
- The predominance of method agreement studies restricted the evaluation of clinical effectiveness, patient outcomes and care pathway integration.
- The generalisability of findings is limited by the small number of studies from low-resource settings.
- Future research should assess multimodal diagnostic pathways and longitudinal outcomes to better inform policy and practice.

## Introduction

Glaucoma is a progressive optic neuropathy and one of the leading causes of irreversible blindness globally. It affects an estimated 60.5 million people worldwide, a figure projected to rise to 111.8 million by 2040, with a disproportionately high burden in African and Asian populations.^1 2^ The disease progresses silently in most cases until advanced stages, resulting in significant visual field loss. Early detection and continuous monitoring are critical to preventing blindness; however, these processes rely on advanced diagnostic technologies and skilled professionals that are often inaccessible in low-resource settings.^3 4^

### Challenges in glaucoma detection and monitoring

The management of glaucoma involves measuring intraocular pressure (IOP), evaluating structural damage to the optic nerve and assessing functional deficits using visual field tests. Goldmann Applanation Tonometry (GAT) is considered the gold standard for IOP measurement, while advanced imaging modalities like optical coherence tomography (OCT) and static automated perimetry are integral for structural and functional assessments.^5 6^ However, such technologies are expensive, require specialised infrastructure and demand highly trained personnel, limiting their availability to urban and tertiary healthcare centres.^7 8^ In low-and middle-income countries (LMICs), the challenges of diagnosing glaucoma are compounded by the lack of primary eye care services, insufficiently trained professionals and limited availability of high-tech equipment.^4 9^ This has led to a significant proportion of individuals remaining undiagnosed until the disease has progressed to advanced stages, contributing to the burden of irreversible blindness.

### The role of portable devices for glaucoma screening and monitoring

Portable devices have emerged as promising tools to address these challenges of diagnosis and monitoring of glaucoma. These devices, which are compact, battery-operated and user-friendly, offer the potential to bring diagnostic and monitoring capabilities to community and primary healthcare settings.^8 10^ Examples include handheld tonometers, portable fundus cameras for optic nerve imaging and smartphone-based visual field analysers.^5 10 11^ Studies have demonstrated that these tools have comparable accuracy to conventional methods, making them valuable for use in remote and resource-limited environments.^12 13^ In high-income countries, portable devices are being experimented on for home monitoring, enabling patients to perform self-assessments of IOP and visual fields.^14 15^ Portable OCT systems using advanced imaging technology have been experimented on to capture detailed retinal images, enabling early detection and monitoring of eye diseases.^16^

### The role of portable devices in eye health

The use of portable devices is not restricted to glaucoma but is also being used in other eye diseases like diabetic retinopathy. These technologies have been used to enhance screening, diagnosis and monitoring of eye diseases, particularly in low-resource and underserved settings. Mobile-based visual acuity applications offer cost-effective and user-friendly alternatives to conventional methods. These tools can be used by non-specialist personnel and can be integrated into community outreach and telemedicine services. The Portable Eye Examination Kit, a smartphone-based application developed by Peek Vision to assess visual acuity, is used in over 75 countries. This exemplifies how smartphone-based platforms can expand access to care in remote areas.^17^,^20^

Portable handheld tonometers, which do not require anaesthesia and can be used in various positions for the comfort of patients, are now available. They are convenient for use in diverse clinical settings such as emergency departments and operating theatres. In addition, portable fundus cameras have been shown to produce high-resolution retinal imaging without the need for pupil dilation and are compatible with Artificial intelligence (AI) systems and electronic health records, enhancing remote diagnosis. Countries, including Finland, Canada and Tasmania, have successfully incorporated these technologies into rural telehealth and mobile clinics, demonstrating improved early detection and continuity of care for conditions such as glaucoma and diabetic retinopathy. Hence, portable devices offer an effective, scalable solution for addressing gaps in eye care delivery, and their adoption holds considerable promise for reducing avoidable vision loss globally.^21^,^33^

In order to investigate these portable devices further, a scoping review methodology was chosen to accommodate the wide range of device types, technologies and outcome measures found in the literature. This approach enables a broad mapping of the evidence base and helps identify areas suitable for future studies.

### Aim

The aim of this scoping review is to identify portable devices used for diagnosing and monitoring glaucoma.

Our research questions included:

1. What is the extent of published literature on the use of portable devices in the detection/diagnosis and monitoring of glaucoma?
2. What is the range of reported agreement, correlation and specificity and sensitivity of these portable devices in detecting/diagnosing and monitoring glaucoma?
3. What can we learn from authors’ reflections on the use of these portable devices?

## Methods

A scoping review protocol was prepared in advance (see online supplemental file).^1^ The review was conducted in accordance with the Joanna Briggs Institute (JBI) Manual for Evidence Synthesis and is reported following the Preferred Reporting Items for Systematic reviews and Meta-Analyses extension for Scoping Reviews (PRISMA-ScR) guidelines.^34 35^

### Inclusion criteria

These include:

- Studies were included if they involved the use of portable devices in any healthcare setting to detect/diagnose and/or monitor glaucoma in adults.
  In our review, we defined ‘portable devices’ as hand-held, battery-operated equipment that are compact, easy to transport and require minimal storage space. By ‘compact’, we refer to devices small enough to be carried by a single person without difficulty and needing specialised containers or trolleys. ‘Easy to transport’ implies that devices can be moved between or within facilities (eg, outreach sites) without needing permanent installation or specialised vehicles. ‘Minimal storage space’ means that the device can be stored in regular shelving, drawers or small storage rooms, unlike larger equipment such as full-sized perimeters or slit lamps. ‘User-friendly’ is not part of the core definition, but was considered a desirable characteristic when discussing feasibility in field settings.
- Studies conducted globally across all regions and ethnic groups were considered eligible for inclusion in this review.
- Original research (as opposed to reviews, editorials or commentaries).

### Exclusion criteria

- Studies in paediatric age groups were excluded from this scoping review.

### Search strategy

A comprehensive literature search was conducted in MEDLINE, Embase, Cochrane Library and Global Health databases from inception to the present. Keywords included “portable glaucoma devices”, “handheld tonometers”, “mobile perimetry” and “telemedicine glaucoma”. Additional studies were identified from reference lists of relevant publications (online supplemental appendix 1).

### Selection of studies

Identified records were imported into Covidence (Covidence systematic review software, Veritas Health Innovation, Melbourne, Australia, available at www.covidence.org) for screening. Title and abstract screening was conducted independently by pairs of reviewers (FG, WN and VHH), with each record reviewed by two authors. Discrepancies were resolved through discussion and consensus. For full-text screening, articles were independently assessed by pairs of reviewers (FG, JE, SHA and ZK) to determine eligibility for inclusion in the data extraction phase. Any disagreements were resolved by discussion and consensus.

### Data extraction

A data extraction template was designed in Covidence software based on the scoping review questions and was piloted by two reviewers (FG and JE). Corrections were made on the data extraction template based on discussion and input from other authors (WN, FK, VHH and MJB) and subsequently adopted prior to extraction. Data extraction was then carried out for each publication independently by five reviewers working in pairs (FG, JE, SHA, ZK and MB). After extraction, all differences were resolved by discussion and consensus.

Studies published in languages other than English were initially screened based on their English titles and abstracts. Where the content appeared relevant, reviewers sought assistance from translation tools (such as Google translator and ChatGPT) to assess eligibility. Data from eligible studies that were successfully translated were extracted. However, scanned articles were not successfully translated. Therefore, data were collected from the English title and abstract.

Data extracted from eligible studies are shown in box 1.

Box 1 **Data extracted from eligible studies**

**General information**

- Study ID.
- Lead author and year.
- Title of paper/abstract/report that data are extracted from.
- Year of publication.
- Corresponding author’s contact details.
- Continent(s) and country(s) in which the study was conducted.

**Aim/purpose**

**Methods**

- Type of study.
- Duration of study (months).
- What outcome(s) was/were measured?
- Type of glaucoma.

**Characteristics of the intervention**

- Main function of portable device(s).
- Description of portable device(s).
- Description of comparison device(s).
- Positions of IOP measurements.

**Characteristics of the study population**

- Total number of participants/sample size.
- % female.
- Age mean, range, %.
- Ethnicity of the study population.
- Health status of study eyes.

**Study findings**

- Study results.
- Author’s conclusion.

IOP, intraocular pressure.

### Study design classification

In this scoping review, we included a range of study designs (table 1) relevant to the evaluation of portable devices for glaucoma diagnosis and monitoring. These included:^36^

**Table 1:** Studies that employed iCare tonometer and comparators (n=101)

Portable device	Comparator	Study type	n (%)	Findings
iCare	GAT	Method agreement	77 (76)	iCare generally agrees well with GAT, with slight underestimation/overestimation. Variability increases at high IOP.
iCare	Perkins	Method agreement	9 (8.9)	iCare matches Perkins closely, within ±2–3 mm Hg. Easier to use in uncooperative patients.
iCare	Others (iCare, TonoPen, Kowa hand-held applanation tonometer or NCT)	Method agreement	7 (6.9)	iCare agrees with other tonometers in normal IOP; variability increases at high IOP. Device-specific biases noted.
iCare	No comparator	Cross-sectional	3 (3.0)	Feasible and reliable for community screening. Easy to use in low-resource settings.
iCare	GAT	Diagnostic test accuracy	1 (1.0)	iCare HOME shows good agreement with GAT, underestimates at low and overestimates at high IOP.
iCare	PAT	Non-randomised controlled trial	1 (1.0)	iCare matches Perkins well across a wide age range. Suitable for diverse age groups.
iCare	GAT	Retrospective	1 (1.0)	iCare doubled detected glaucoma cases with a modest screening increase, proving valuable in Africa.
iCare	GAT	Prospective observational	1 (1.0)	Drive-through IOP with iCare overestimates by 2.4 mm Hg on average. Promising for high-throughput screening.
iCare	GAT	Meta-analysis	1 (1.0)	Meta-analysis showed no significant mean difference versus GAT, but high heterogeneity across studies.

GAT, Goldmann Applanation Tonometry; IOP, intraocular pressure; NCT, Non-Contact Tonometer; PAT, Perkins Applanation Tonometry.

- Method agreement studies, which assess how well a new device or method agrees with a reference standard (often reported using statistical tools such as Bland-Altman plots or intraclass correlation coefficients).
- Diagnostic accuracy test studies, which evaluate the sensitivity, specificity and predictive values of a test compared with a gold standard.
- Feasibility or usability studies, which examine the practical use, acceptability and user experience of the device in clinical or community settings.
- Validation studies, which determine whether a device produces valid and reliable measurements under specified conditions.
- Descriptive and cross-sectional studies, which provide snapshots of device performance or usage without testing a specific hypothesis.

### Synthesis of the results

We conducted a descriptive analysis of the study characteristics, study methods and of the main function of the portable device used, the portable device used in the study and the comparison equipment. Study characteristics included the location of the study, year of publication, average age, percentage female population and ethnicity of participants in the study.

## Results

A total of 1441 records were identified through searches of various databases and registers. From this total, 697 references were removed due to duplication (15 duplicates were identified manually and 682 were identified automatically by Covidence). This step reduced the dataset to 744 records for further screening.

At the screening stage, based on titles and abstracts, 510 records were excluded as they did not meet the inclusion criteria.

- Studies with no use of portable device: 230 (45.1%).
- Studies not evaluating portable devices or glaucoma: 163 (32.0%).
- Studies involving paediatric populations: 95 (18.6%).
- Not original research: 22 (4.3%).

The remaining 233 records were deemed suitable for full-text review, with almost all articles successfully retrieved for assessment except for 3 articles, which could not be retrieved.

**Figure 1:**
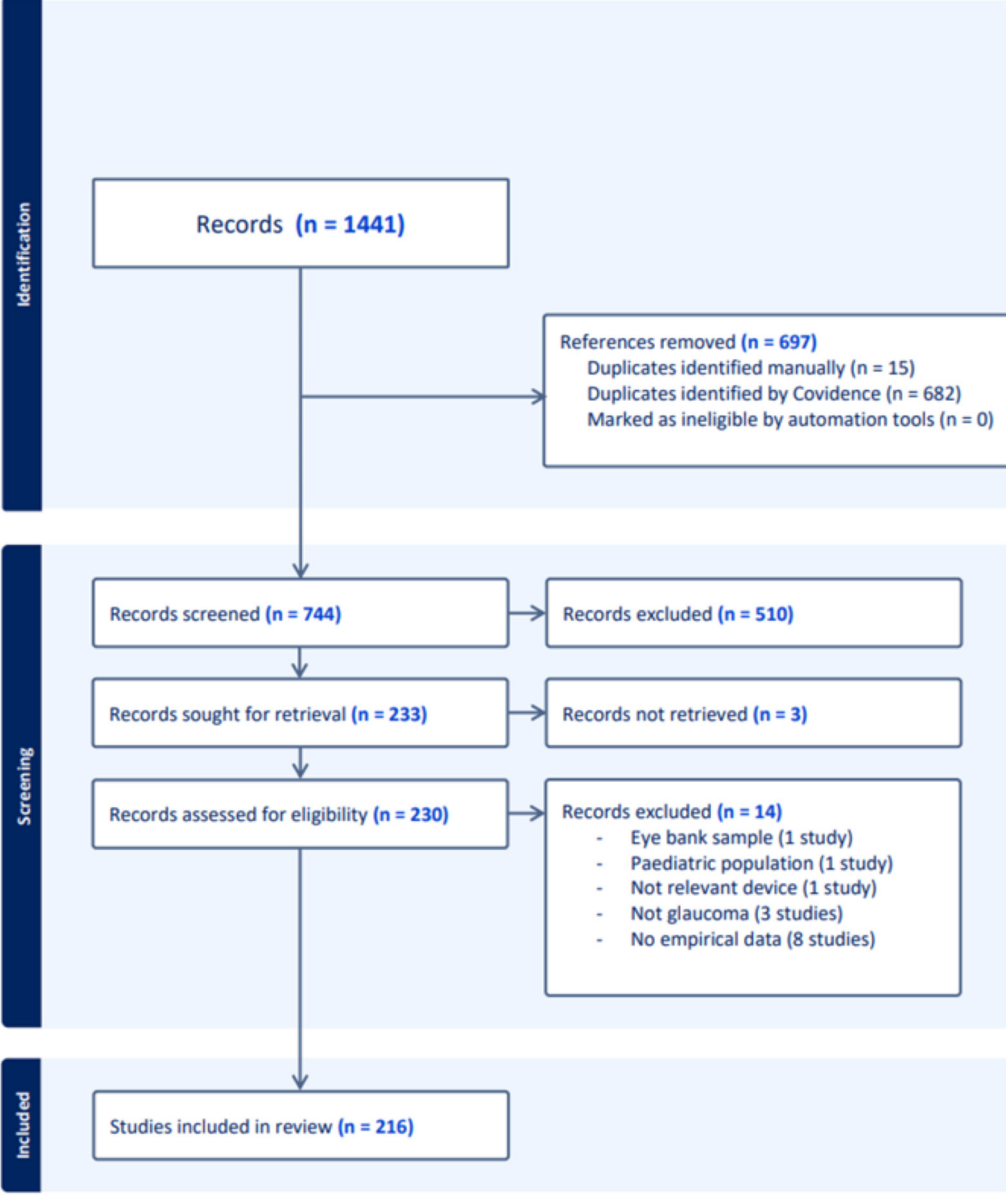
Preferred Reporting Items for Systematic Reviews and Meta-Analyses (PRISMA) 2020 flow diagram. Portable devices for the detection, diagnosis and monitoring of glaucoma.

Among the 230 studies assessed for eligibility, 14 were excluded as shown in figure 1 and online supplemental table, appendix II. This left 216 studies that met the criteria for inclusion and were included in the review, representing the body of evidence used for analysis and synthesis. Figure 1 shows a PRISMA flowchart, which visually conveys the stepwise reduction in the number of studies. This structured methodology aligns with JBI Manual for Evidence Synthesis and PRISMA guidelines, emphasising transparency and replicability in the review process.^34 35^

The studies reviewed included a wide range of participant demographics (see online supplemental table, appendix III). The median sample size was 85, with study populations ranging from 21 to 4550 participants, indicating both small-scale and large-scale investigations. The median percentage of female participants was 56%, with a range of 8%–95%, reflecting varying levels of gender representation. The median average age of participants was 55 years, with ages spanning from less than 1 year to 95 years, suggesting inclusion of both younger adults and older populations across the studies. Although this review excluded studies focused on paediatric populations, one study was identified that included both children and adults. As adults comprised 90% of the total study population, a decision was made to retain the study within the review due to its relevance and applicability to the adult population.^37^

**Figure 2:**
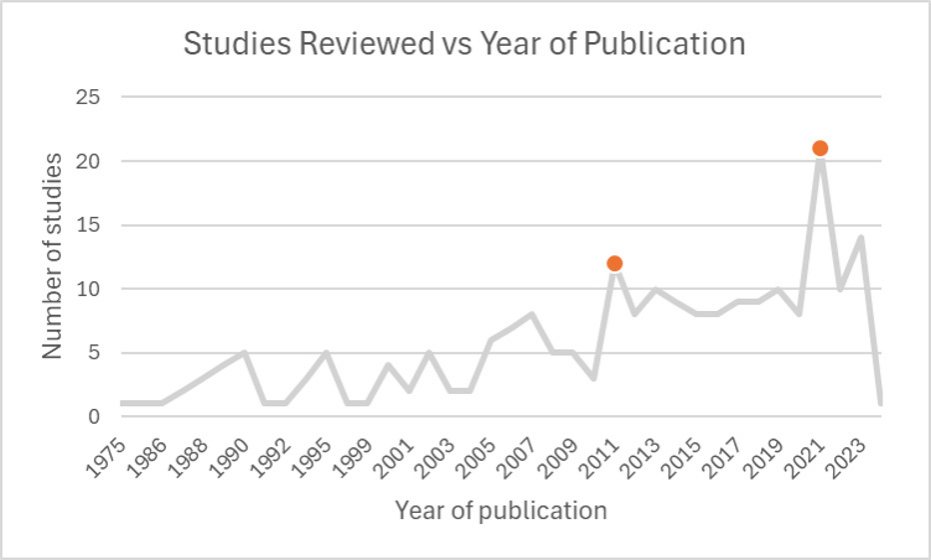
Line chart displaying the distribution of studies by the year of publication.

Figure 2 shows the extent of published literature on portable devices for glaucoma by year of publication. The number of studies remained low and irregular from the late 1970s until around 2010, after which a notable increase in publications was observed. The highest number of studies was published in 2020, with some variation in subsequent years and a drop in 2023.

Table 2 summarises the distribution of studies across different continents, with a total of 216 studies conducted globally. It highlights the geographic representation of research, showing varying levels of contribution from each continent.

**Table 2:** Distribution of studies by continent

Continent in which the study was conducted	Count	Percentage (%)
North America	62	28.7
Asia	58	26.9
Europe	56	25.9
Oceania	23	10.6
Africa	11	5.1
South America	4	1.9
Multicontinental	2	0.9
Grand total	**216**	**100**

A wide range of portable ophthalmic devices with various functions was identified in this review (see online supplemental table, appendix IV). These devices were categorised into four main groups: visual acuity assessment tools, portable tonometers for IOP measurement, visual field testing devices and handheld fundus cameras for retinal imaging.

The majority of studies (81%) focused on evaluating IOP measuring devices. Devices for visual field assessment accounted for 12.5% of the studies, while those for fundus examination comprised 5%. Only one study (0.5%) combined the use of an IOP measuring device with a fundus examination device. Only two studies (0.9%) evaluated devices that included IOP measurement, visual field testing and fundus imaging together.

Table 3 summarises the distribution of study types reviewed. Method agreement studies dominate the dataset, accounting for 195 studies, which represent 90.3% of the total 216 studies. In contrast, other studies, comprising a mix of prospective observational studies, cross-sectional descriptive studies, diagnostic accuracy studies, a meta-analysis, a non-randomised controlled trial and a qualitative study, account for only 21 studies (9.7%). This striking imbalance highlights the overwhelming focus on method agreement assessments within the portable device literature, while other study designs remain relatively under-represented but contribute important complementary perspectives.

**Table 3:** Type of studies reviewed

Grouped type of study	Number of studies	Percentage
Method agreement	195	90.3
Prospective/observational	8	3.7
Cross-sectional/descriptive	8	3.7
Diagnostic accuracy	2	0.9
Meta-analysis	1	0.5
Non-randomised controlled trial	1	0.5
Qualitative study	1	0.5
Total	**216**	**100**

In table 1, a variety of study designs that assessed the use of the iCare tonometer across clinical and screening settings are presented. Of the 101 studies reviewed that employed iCare, 91 studies (90%) were method agreement studies. The most frequently used comparator with iCare is GAT, followed by Perkins, other iCare models, TonoPen and Non-Contact Tonometers. Cross-sectional and observational studies examined its application in large-scale or low-contact settings, such as community outreach programmes and drive-through clinics.

In table 4, a summary of studies assessing portable visual field testing devices is presented; organised by study type and comparator. The method agreement studies category includes Melbourne Rapid Fields (MRF) and Eyecatcher, with study population, demographic characteristics and key findings reported. The diagnostic test accuracy studies list MRF-S and Easy Perimetry, detailing total participants, average age and performance metrics such as AUC and sensitivity. The qualitative study describes participant characteristics and reported experiences with Eyecatcher for home use. The cohort study outlines findings from weekly home monitoring using MRF, including participant demographics and test outcomes.

**Table 4:** Studies that employed visual dield devices and comparators (n=27)

Portable device	Comparator	n (%)	Characteristics	Findings
*Method agreement studies*				
Melbourne Rapid Fields (MRF)	Humphrey Field Analyzer (HFA)	9 (33.3)	Median sample average for study population: 60.0 (range 28–143) Median % female: 48% (range 26–83) Median average age: 57 years (range 24–69)	MRF tests, particularly on tablets, demonstrate good agreement with HFA, offering reliable, efficient and repeatable visual field assessments. While MRF may slightly overestimate or underestimate certain parameters and is less effective at detecting early glaucoma, it remains a viable and scalable option for home or low-resource settings.
Eyecatcher	HFA	3 (11.1)	Median sample average for study population: 64 (range 20–77) Median %female: 50% (range 43–67%) Median average age: 59 years (range 24–71)	Eyecatcher visual field test demonstrated good agreement with standard perimetry, high user acceptability and reliable performance in both clinical and home settings. It shows promise as a practical tool for triaging in clinics and home monitoring.
*Diagnostic test accuracy study*				
MRF	Octopus HFA	1 (3.7)	Total participants: 142 Average age: 67	MRF-S iPad-based test demonstrated strong diagnostic accuracy for detecting moderate (AUC=0.87) and mild (AUC=0.81) visual field defects, with significantly shorter test duration (1.88 vs 5.92 min) and high user acceptability.
Easy perimetry	HFA	1 (3.7)	Total participants: 115 % female: 42% Average age: 65 years	Easy perimetry app showed limited diagnostic performance, with an AUROC curve of 0.64 for any visual field defect and 0.68 for moderate or worse glaucoma. At 90% specificity, the sensitivity was only 35%, indicating the tool lacks sufficient accuracy for effective glaucoma screening in this population.
*Qualitative study*				
Eyecatcher	HFA	1 (3.7)	Total participants: 20 % female: 50% Median age: 71 years (range 62–78)	Participants were able to complete home visual field (VF) testing using the Eyecatcher system. Participants generally viewed home monitoring favourably compared with standard clinic-based testing, highlighting benefits such as ease of use and convenience.
*Cohort study*				
MRF	HFA	1 (3.7)	Total participants: 43 Average age: 71 years (range 37–89)	Weekly home monitoring detected rapid visual field loss much earlier (0.9 years) than 6-monthly clinic testing (2.5 years), even with moderate compliance (63%). This suggests that home monitoring may significantly improve early detection of glaucoma progression, although its cost-effectiveness remains to be evaluated.

AUC, Area Under the Curve

AUROC,Area Under the Receiver Operating Characteristic

AUC, Area Under the Curve; AUROC, Area Under the Receiver Operating Characteristic .

A summary of studies assessing portable fundus cameras, also organised by study type and comparator, is shown in table 5. The method agreement studies include devices such as the Nidek, Volk Pictor and Optain cameras, with reported sample sizes, demographic characteristics and agreement statistics, including kappa and Intraclass Correlation Coefficient (ICC) values. The only diagnostic test accuracy study in this group evaluated multiple portable devices, namely, Remidio, Pictor Plus, iNview and oDocs visoScope. These devices were compared against a table-top Zeiss fundus camera. Details of participant characteristics and findings were also captured in table 5.

**Table 5:** Studies that employed fundus imaging devices and comparators (n=11)

Portable device	Comparator	n (%)	Characteristics	Findings
*Method agreement studies*				
Nidek	Zeiss FF retinal camera	3 (27.3)	Median sample average for study population: 27 (range 14–27)	Nidek fundus camera showed moderate to good agreement for optic disc assessment with kappa values of 0.49–0.52 and sensitivity/specificity ranging from 67% to 87% and 68% to 79%, respectively, while agreement with gold-standard photographs ranged from 0.45 to 0.87.
Volk Pictor	Slit-lamp CDR/DDLS grading Topcon table-top mydriatic fundus camera	2 (18.2)	Average study population: 115 (range 119–211) Average % female: 52.0% Average age: 59 years (Range 24–71)	Volk Pictor camera provides moderate intra- and interobserver agreement (ICC 0.65–0.71; κ=0.54–0.64) and good diagnostic accuracy (AUC up to 0.88) for optic disc evaluation, though image quality was often suboptimal (only 8%–32% rated as ‘good’). In comparison, Topcon fundus photography showed slightly higher inter-observer and intra-observer agreement (κ up to 0.80), and no significant differences in cup-to-disc ratio (CDR) measurements were observed between the devices, suggesting both may be viable for glaucoma screening, with room for improvement in imaging consistency and quality.
Optain OPTFC01 camera	Topcon table-top mydriatic fundus camera	1 (9.1)	Total participants: 504	Results show poor consistency for glaucomatous optic neuropathy (κ=0.32).
*Diagnostic test accuracy study*				
Remidio NMFOP Volk Pictor Plus Volk iNview oDocs visoScope	Zeiss Visucam	1 (9.1)	Total participants: 28 Average age: 41	Remidio and Pictor handheld fundus cameras performed comparably to the Zeiss table-top device in image acquisition, quality and diagnostic sensitivity. Their high gradeability and reliable cup-to-disc ratio estimates support their use as practical, cost-effective tools in remote or resource-limited clinical environments.

## Discussion

This scoping review highlights a growing and diverse body of literature evaluating the use of portable devices for the detection, diagnosis and monitoring of glaucoma. With 216 studies included, the review provides a comprehensive overview of available portable technologies, their performance characteristics and the contexts in which they have been deployed. The overwhelming predominance of method agreement studies (90.3%) indicates a sustained focus on validating new or portable devices against established gold standards such as GAT, Humphrey Field Analyzer (HFA) and tabletop fundus cameras. While fewer studies employed diagnostic accuracy designs, qualitative assessments or prospective evaluations, these studies contributed critical insights into feasibility, usability and patient-centred outcomes.

Across the different device categories, namely tonometers, visual field analysers and fundus cameras, several key themes emerged. The iCare tonometer, used in 97 studies (45% of all included), was consistently reported to provide IOP measurements within clinically acceptable limits when compared with GAT, Perkins and other portable tonometers. The iCare device also featured prominently in novel settings such as drive-through clinics and community screening, where its portability and ease of use were especially advantageous.

In the area of portable visual field testing, MRF and Eyecatcher were the most frequently studied devices. Method agreement studies dominated this category, with the HFA being the most commonly used comparator. These devices were primarily employed for detecting mild to moderate glaucomatous visual field defects. Eyecatcher was evaluated in both clinical and home settings, with a focus on its potential use in triaging and monitoring individuals at risk of glaucoma.

Fundus imaging devices accounted for a smaller proportion of the identified studies. Devices such as the Volk Pictor and Nidek cameras were assessed for agreement with standard fundus photography in measuring optic disc features like vertical cup to disc ratio. The Optain camera was evaluated in a larger study to assess its function in detecting glaucomatous optic neuropathy. Remidio and Pictor Plus were included in diagnostic test accuracy studies and examined in the context of teleophthalmology and AI integration in remote settings.

The global distribution of studies, spanning all continents with the highest representation from North America, Asia and Europe, illustrates increasing international interest in the use of portable tools for glaucoma care. Nevertheless, regions such as Africa and South America remain under-represented, despite having high glaucoma burdens and lower access to conventional diagnostic services. With a median average age of 55 years in the reviewed studies, there was an under-representation of studies evaluating these tools in individuals with mobility issues, nursing homes, home settings or remote/rural populations and those in underserved settings. These group of people will benefit more from the use of portable devices.

The temporal trend of publications also showed a surge in studies post-2010, peaking in 2020. Possible reasons behind the acceleration of research in this field during these periods could be due to technological innovation fuelled by advances in miniaturisation, the need to improve convenience, the development of wireless communication and AI, which have enabled more sophisticated portable devices. Patient empowerment and telemedicine trends through growing interest in home-based monitoring and self-testing, especially among tech-literate populations and individuals with disabilities, could also be another reason for the progress in this field. The ageing populations and workforce shortages, coupled with the COVID-19 pandemic, have created pressures in the health systems. This has also prompted efforts to decentralise eye care and triage more effectively at the primary level, further catalysing interest in remote diagnostics.

Importantly, the diversity of devices and study methodologies captured in this review underscores the need for standardised reporting frameworks. A large number of studies did not report essential demographic variables such as ethnicity or the detailed health status of study eyes or the types of glaucoma evaluated. These gaps limit the generalisability and utility of findings, particularly in under-resourced settings where population characteristics may significantly influence device performance. Many agreement studies failed to mention participants’ prior exposure to the reference standard (eg, HFA), potentially giving them an advantage compared with unfamiliar comparator devices. The learning curve of new or portable devices should be accounted for in the studies. As a result, reported agreement may not be accurate, particularly for perimetry measurements, which require users’ actions, feedback or alignment. Future studies should assess test-retest reliability across different levels of user experience.

Only two studies included in the review assessed all three categories of portable devices within the same investigation. Comprehensive glaucoma assessment typically requires the integration of these three components to support accurate diagnosis and effective monitoring. The limited number of studies evaluating all modalities simultaneously highlights a gap in the current literature, particularly in the context of LMICs, where integrated, resource-efficient approaches are essential. The practical advantages of portable, battery-operated, low-maintenance devices in areas lacking fixed infrastructure seem promising. Coupled with its importance of co-developing workflows with community health workers and non-ophthalmic personnel. This underlines the need for future real-world implementation research to explore combined diagnostic pathways using multiple portable tools, especially in LMIC contexts, as proof-of-concept. Data alone may not reflect feasibility or scalability.

The authors’ reflections across the studies vary but generally focus on comparing portable devices to gold standard methods for measuring IOP. It is also important to note that earlier studies often lacked standardised criteria for glaucoma diagnosis (eg, predating International Society of Geographical and Epidemiological Ophthalmology). Device types have also evolved significantly (from analogue tonometers to digital, AI-assisted platforms). It is therefore not unexpected that the outcomes and reference standards used have varied across time. Several conclusions also emphasise the potential of these devices for use in screening or primary care settings, with recommendations for further refinement or context-specific application. The general consensus supports the complementary use of portable devices in the detection and monitoring of glaucoma while acknowledging that they may not fully replace standard equipment in all clinical scenarios.

The scope of this review was shaped by methodological choices inherent to scoping review design. We prioritised breadth of evidence capture over critical appraisal, which meant that grey literature and trial registries were not systematically searched, and a formal risk of bias assessment was not undertaken. While this approach strengthens inclusivity, it also limits certainty about the robustness of individual studies and the overall evidence base.

The available literature further constrained interpretation. Most studies were limited to method agreement, offering useful technical comparisons but providing little insight into clinical effectiveness, patient outcomes or pathways of care. Evidence from low-resource settings was sparse, reducing the generalisability of findings to contexts where portable devices may be most transformative. These limitations highlight the need for future research that goes beyond technical validation to evaluate portable devices within multimodal diagnostic strategies and over longer periods, generating evidence that can guide practice and policy.

## Conclusion

Portable glaucoma devices are demonstrating increasingly robust performance and versatility across a range of clinical and non-clinical environments. While most evidence is focused on method agreement, emerging data from diagnostic, qualitative and implementation studies offer valuable insights into real-world utility. Future research should aim to expand geographical coverage, incorporate more robust study designs and explore the integration of these tools into comprehensive eye care pathways, especially in underserved communities. Their potential for enhancing accessibility, early detection and continuity of care makes portable devices a promising solution for addressing the global burden of glaucoma.

## Supplementary material

10.1136/bmjopen-2025-105681 online supplemental file 1

10.1136/bmjopen-2025-105681 online supplemental file 2

10.1136/bmjopen-2025-105681 online supplemental file 3

10.1136/bmjopen-2025-105681 online supplemental file 4

10.1136/bmjopen-2025-105681 online supplemental file 5

## Data Availability

Data sharing not applicable as no datasets generated and/or analysed for this study.
