# Supplementary material for: Portable devices for the diagnosis of glaucoma: a scoping review
Source: BMJ Open. 2025 Oct 21;15(10):e105681. doi: 10.1136/bmjopen-2025-105681 (PMC12548591; doi:10.1136/bmjopen-2025-105681)
Supplement: online supplemental file 1 [file bmjopen-15-10-s001.docx]

**Appendix I: MEDLINE search strategy.**

MEDLINE Ovid

1. exp Glaucoma/

2. Intraocular Pressure/

3. Ocular Hypertension/

4. Visual Field Tests/

5. (glaucoma$ or ocular hypertensi$).tw.

6. intraocular pressure$.tw.

7. intra-ocular pressure$.tw.

8. (visual adj2 field$ adj2 test$).tw.

9. (retina$ adj2 imaging).tw.

10. (OAG or POAG or ACG or PACG or IOP or OHT).tw.

11. or/1-10

12. EyeSnellen.tw.

13. ((EyeChart or EyeXam) adj1 app).tw.

14. (Peek adj1 acuity).tw.

15. iSight Pro.tw.

16. SmartOptometry.tw.

17. ((handheld or hand-held or portable or smartphone or app) adj2 (Snellen or

LogMAR)).tw.

18. ((handheld or hand-held or portable or smartphone or app) adj2 visual acuit$).tw.

19. (Peek adj1 contrast adj1 sensitivity).tw.

20. PeekCS.tw.

21. VCS Test online.tw.

22. (ICare adj4 tonometer$).tw.

23. (Perkin$ adj4 tonometer$).tw.

24. Tonopen.tw.

25. ((handheld or hand-held or portable) adj4 tonometer$).tw.

26. (Topcon adj3 NW8).tw.

27. (Remidio adj2 NMFOP).tw.

28. (Volk adj3 (Pictor or iNview)).tw.

29. (oDocs adj2 visoScope).tw.

30. (MiiS adj2 Horus adj2 Scope).tw.

31. (Horus adj3 (DEC200 or DEC300)).tw.

32. Smartscope.tw.

33. ((handheld or hand-held or portable) adj4 (fundus adj2 camera)).tw.

34. (Portable adj2 slit adj2 lamp).tw.

35. Eyecatcher.tw.

36. ((tablet or ipad) adj1 perimeter$).tw.

37. (Melbourne adj1 Rapid adj1 Fields).tw.

38. (VF2000 adj1 Focus).tw.

39. ((handheld or hand-held or portable) adj4 visual adj1 field$).tw.

40. (portable adj2 perimetry).tw.

41. nGoggle.tw.

42. (C3 adj2 field adj2 analyzer$).tw.

43. C3FA.tw.

44. (VisualFields adj1 Easy).tw.

45. (Visual adj1 Fields adj1 Easy).tw.

46. (virtual adj1 reality adj1 glasses).tw.

47. or/12-46

48. 11 and 47

49. Animals/

50. (animal or animals or mouse or mice or rat or rats or rabbit$ or dog or dogs or canine

or cat or cats or pig or pigs or veterinary).tw.

51. or/49-50

52. 48 not 51

53. exp case reports/

54. (case adj2 report$).tw.

55. or/53-54

56. 52 not 55
