# Supplementary material for: Portable devices for the diagnosis of glaucoma: a scoping review
Source: BMJ Open. 2025 Oct 21;15(10):e105681. doi: 10.1136/bmjopen-2025-105681 (PMC12548591; doi:10.1136/bmjopen-2025-105681)
Supplement: online supplemental file 2 [file bmjopen-15-10-s002.docx]

**Appendix II: Excluded Studies and Reason for Exclusion**

| **Citation Label** | **Title** | **Authors** | **Country** | **Reason for Exclusion** |
| --- | --- | --- | --- | --- |
| Odayappan 2023 | Comparison of a New Head Mount Virtual Reality Perimeter (C3 Field Analyzer) With Automated Field Analyzer in Neuro-Ophthalmic Disorders | Odayappan, Annamalai et al. | United States | Wrong outcomes |
| Briesen 2010 | Minimal cross-infection risk through iCare rebound tonometer probes | Briesen, S et al. | England | Wrong outcomes |
| Nakakura 2018 | iCare R rebound tonometers: review of their characteristics and ease of use | Nakakura, Shunsuke | New Zealand | Wrong study design |
| Whitacre 1991 | The effect of Perkins, Tono-Pen, and Schiotz tonometry on intraocular pressure | Whitacre, M M et al. | United States | Wrong setting |
| Cook 2012 | Systematic review of the agreement of tonometers with Goldmann applanation tonometry | Cook, Jonathan Alistair et al. | United States | Wrong study design |
| Burdova 2011 | Correlation of IOP measured by applanation, noncontact tonometry and TonoPen with CCT | Burdova, M Ceska et al. | Czech Republic | Wrong outcomes |
| Wingert 1995 | Clinical evaluation of five portable tonometers | Wingert, T A et al. | United States | Wrong study design |
| Perkins 1965 | Hand-held applanation tonometer | Perkins, E S | England | Wrong study design |
| Armstrong 1990 | Evaluation of the Tono-Pen and the Pulsair tonometers | Armstrong, T A | United States | Wrong study design |
| Palmer 2018 | Glare-free retinal imaging using a portable light field fundus camera | Palmer, Douglas W et al. | United States | Wrong study design |
| Wasielica-Poslednik 2015 | Comparison of Rebound Tonometry, Perkins Applanation Tonometry and ORA in MPS Patients | Wasielica-Poslednik, Joanna et al. | United States | Paediatric population |
| Damato 1994 | Multifixation campimetry: Recent developments | Damato, B.E. | United States | Wrong study design |
| Wei 2023 | Hybrid spiral scanning in a double-clad fibre-based handheld confocal scanning light ophthalmoscope | Wei, Franklin et al. | United States | Wrong study design |
| Realini 2021 | Assessing the Reliability of Intraocular Pressure Measurements Using Rebound Tonometry. | Realini, Tony et al. | United States | Wrong study intervention |
