# Supplementary material for: Portable devices for the diagnosis of glaucoma: a scoping review
Source: BMJ Open. 2025 Oct 21;15(10):e105681. doi: 10.1136/bmjopen-2025-105681 (PMC12548591; doi:10.1136/bmjopen-2025-105681)
Supplement: online supplemental file 3 [file bmjopen-15-10-s003.docx]

**Appendix III: Study Population Characteristics Summary**

| **Characteristics** | **Median** | | **Range (Min - Max)** |
| --- | --- | --- | --- |
| Sample Size | | 85 | 21 - 4550 |
| % Female | | 56 | 8 - 95 |
| Average Age (Years) | | 55 | <1 - 95 |
