# Supplementary material for: Portable devices for the diagnosis of glaucoma: a scoping review
Source: BMJ Open. 2025 Oct 21;15(10):e105681. doi: 10.1136/bmjopen-2025-105681 (PMC12548591; doi:10.1136/bmjopen-2025-105681)
Supplement: online supplemental file 4 [file bmjopen-15-10-s004.docx]

**Appendix IV: Portable Devices Identified in Reviewed Studies**

| **Category of Portable Devices** | **Portable Devices in the Review** |
| --- | --- |
| **Visual Acuity Assessment** | **“E”printed card** |
| **Tonometers** | **Applanation Tonometers** |
|  | Goldmann Applanation Tonometer (standard reference) |
|  | Perkins Applanation Tonometer |
|  |  |
|  | **iCare Rebound Tonometer** |
|  | iCare ic100 |
|  | iCare IC200 |
|  | iCare TA01i |
|  | iCare Home |
|  | iCare Home2 |
|  | iCare PRO |
|  |  |
|  | **Non-contact Tonometers (Air-puff)** |
|  | Pulsair IntelliP |
|  | Pulsair CT-10 |
|  | Topcon CT-800A |
|  | Topcon CT-80 |
|  | Reichert AT550 |
|  | Keeler Non-Contact Tonometer |
|  | Noncontact Tonometer CT 60 computerised tonometer |
|  | Corvis ST |
|  |  |
|  | **TonoPen** |
|  | TonoPen XL |
|  | TonoPen TPA |
|  | TonoPen AVIA |
|  |  |
|  | **Dynamic Contour Tonometers (DCT)** |
|  | Pascal DCT |
|  | Hand-held Dynamic Contour Tonometer (HH-DCT) |
|  |  |
|  | **Transpalpebral Tonometers** |
|  | Easyton (TVGD-02) |
|  | TGDc-01 |
|  | Transpalpebral TGDc-01 PRA Tonometer |
|  | Diaton Tonometer |
|  |  |
|  | **Others Tonometers** |
|  | Proview |
|  | Schiotz |
|  | IOPen |
|  | Reichert Ocular Response Analyzer |
|  | Handheld pressure phosphene tonometer |
|  | Proview eye pressure monitor |
|  | The Ocuton-A and Ocuton-S tonometers |
|  | Proton Tonometer (Tomey) |
|  | Microprocessor-controlled self-tonometer |
|  | Keta Smart-001 |
|  |  |
| **Visual Field Testing Devices** | Melbourne Rapid Fields (MRF) iPad-based perimetry software |
|  | Eyecatcher VF tests |
|  | Visual Fields Easy screening |
|  | Oculus VR Kinetic Perimetry |
|  | IMO vifa |
|  | Oculus Quest VR headset with the Bluetooth connected clicker |
|  | The Visual Field Easy app |
|  | 22″ LCD Display with Custom Software |
|  | Virtual reality (VR) glasses Trust EXOS 3D VR glasses and Alcatel One Touch Pixi 4 (6) |
|  |  |
| **Fundus Cameras** | Remidio Non-Mydriatic Fundus camera |
|  | Volk Pictor Plus |
|  | Volk iNview |
|  | Smartscope non-mydriatic fundus camera |
|  | Optain OPTFC01 |
|  | Pictor camera |
|  | PanOpticTM iExaminer |
|  | Nidek NM100 fundus camera |
|  | TMC-63M Fundus camera |
|  | oDocs visoScope |
|  | DIO with a CCD camera connected PCMCIA frame-grabber |
