# Supplementary material for: Portable devices for the diagnosis of glaucoma: a scoping review
Source: BMJ Open. 2025 Oct 21;15(10):e105681. doi: 10.1136/bmjopen-2025-105681 (PMC12548591; doi:10.1136/bmjopen-2025-105681)
Supplement: Supplementary file 6 [file bmjopen-2025-105681.reviewer_comments.pdf]

PEER REVIEW HISTORY

BMJ Open publishes all reviews undertaken for accepted manuscripts. Reviewers are asked to complete a checklist review form and are provided with free text boxes to elaborate on their assessment. These free text comments are reproduced below.

ARTICLE DETAILS

Title (Provisional)

PORTABLE DEVICES FOR DIAGNOSIS OF GLAUCOMA: SCOPING REVIEW

Authors

Garba, Farouk; Kyari, Fatima; Burton, Matthew J; Adler, Samuel Harris; Khan, Zunaira; Bello, Mubarak; Evans, Jennifer; Gordon, Iris; Hu, Victor H.; Nolan, Winifred

---

VERSION 1 - REVIEW

**Reviewer**	**1**
**Name**	**Stagg, Brian**
**Affiliation**	**University of Utah Health**
**Date**	**02-Jul-2025**
**COI**	**None**

This is an excellent review about an important topic--the use of portable devices for diagnosis of glaucoma.

I have a few comments:

1) The definition of "portable devices" could use more specificity. What does compact mean? Is user-friendly (pg 4 line 22) part of the definition? What does easy to transport mean? What does "require minimal storage space" mean? (pg 5 line 43)

2) Pg 4, line 46: I may have missed this, but what is PEEK?

3) PG 8, line 11: 510 studies were excluded as they did not meet the inclusion criteria. I may have missed this, but can you tell us which inclusion criteria they didn't meet?

4) Were any devices excluded because they didn't meet the definition of "portable devices?"

5) PG 12, line 6: Is the 0.9% 2 studies?

6) Appendix III: It seems that some devices may be missing. For example:

Phu J, Wang H, Kalloniatis M. Comparing a head-mounted virtual reality perimeter and the Humphrey Field Analyzer for visual field testing in healthy and glaucoma patients.

Ophthalmic Physiol Opt. 2024 Jan;44(1):83-95. doi: 10.1111/opo.13229. Epub 2023 Oct 6. PMID: 37803502; PMCID: PMC10952716.

Bradley C, Ahmed IIK, Samuelson TW, Chaglasian M, Barnebey H, Radcliffe N, Bacharach J. Validation of a Wearable Virtual Reality Perimeter for Glaucoma Staging, The NOVA Trial: Novel Virtual Reality Field Assessment. Transl Vis Sci Technol. 2024 Mar 1;13(3):10. doi: 10.1167/tvst.13.3.10. PMID: 38488433; PMCID: PMC10946691.

I wonder if the search strategy didn't pick these and others up?

7) Did you find any studies for portable OCT devices? Would your search strategy have picked this up?

**Reviewer**	**2**
**Name**	**Abegão Pinto, Luis**
**Affiliation**	**Centro Hospitalar Lisboa Norte EPE, Department of Ophthalmology**
**Date**	**12-Jul-2025**
**COI**	**None**

The authors have presented a scoping review of portable diagnostic devices/methods in Glaucoma. While interesting and well presented, there are some concerns that should be addressed:

1) a scoping review is done mostly on exploratory work, to clarify existing definitions or to include a wide range of study designs. While it does match the purpose of the study, by including nearly 50 years of study, there is a risk of studies having been too much diverse and using different criteria (from inclusion to outcomes). This should be expanded (and would perhaps highlight further how in recent years the literature is expanding at a faster pace

2) linking to the prior argument, it would be interesting to hear the authors view on why the pace is increasing. Is it a cultural/societal demand for portable devices? patient empowerment? or merely technological development?

3) the populations tested are likely not the ones more likely to be the main target of these. Living in rich countries and in their 50s. The age part seems particularly interesting as the less mobile patients (older) would likely having more difficulties to travel and use this in nursing homes or in remote, underserved areas.

4) I would like to have expanded in the discussion the section of representativity and the possibility of use in underdeveloped areas

%) finally, some studies are agreement studies whose results are probably overestimated since they are comparing subjects already skilled in the use of a certain device (Humphrey

for instance) while likely neglecting to take into the account the learning curve of the comparator. It would be interesting to hear the authors view on this bias and, if required, listed it in the limitations

---

VERSION 1 - AUTHOR RESPONSE

Reviewer 1:

Thank you for the taking the time to review our manuscript. Find responses to you valuable comments below.

1. Definition of "portable devices":(Page 5 , line 1).

In our review, we defined “portable devices” as hand-held, battery-operated equipment that are compact, easy to transport, and require minimal storage space. By “compact,” we refer to devices small enough to be carried by a single person without difficulty and needing specialised containers or trolleys. “Easy to transport” implies that devices can be moved between or within facilities (e.g. outreach sites) without needing permanent installation or specialised vehicles. “Minimal storage space” means that the device can be stored in regular shelving, drawers or small storage rooms, unlike larger equipment such as full-sized perimeters or slit lamps. “User-friendly” is not part of the core definition, but was considered a desirable characteristic when discussing feasibility in field settings.

2. Pg 4, line 46: What is PEEK?

Thank you for pointing this out. We have revised this to include the clarification **(Page 4 , line 1).**

PEEK refers to the Portable Eye Examination Kit, a smartphone-based application developed by Peek Vision to assess visual acuity.

3. Pg 8, line 11: Inclusion criteria not met by 510 studies

The 510 studies were excluded for not meeting our predefined inclusion criteria, which included: **(Page 4 , line 37)**

- Studies were included if they involved the use of portable devices in any healthcare setting to detect/diagnose and/or monitor glaucoma in adults. Portable devices were defined as handheld, battery-operated, compact tools that are easy to transport and require minimal storage space. Examples include the iCare tonometer, Tono-Pen, Perkins applanation tonometer, handheld fundus cameras, and portable visual field analysers.

- Studies conducted globally across all regions and ethnic groups were considered eligible for inclusion in this review.
- Original research (as opposed to reviews, editorials, or commentaries)

Most were excluded because they did not evaluate portable devices, not for assessment of glaucoma, or involved paediatric populations.

4. Devices excluded for not meeting "portable" definition?

We did not encounter any device that were excluded because they did not meet our definition of “portable devices”.

5. Pg 12, line 6: Is 0.9% = 2 studies?

Yes, that is correct **(Page 10 line 14).** The 0.9% figure corresponds to 2 studies out of the total number of 216 studies included in our final synthesis compared intraocular, visual field and optic nerve head assessment devices.

6. Appendix III: Missing devices (e.g. VR perimeters)? (Page 2 , line 28).

Thank you for pointing this out. The two Virtual Reality (VR) perimeter studies you referenced; Phu et al. (2024) and Bradley et al. (2024 were not captured in our initial search. This is because our search strategy did not include emerging VR terminology. We acknowledge this as a limitation and plan to refine and expand our search strategy to include VR terminology in future reviews.

7. Did you find any studies for portable OCT devices? Would your search strategy have picked this up? (Page 2 , line 28).

We did not include OCT-specific terms in our search strategy, and therefore did not identify any studies on portable OCT devices in our final review. We also acknowledge this as a limitation and plan to refine and expand our search strategy to include OCT-related terminology in future updates of the review.

Reviewer 2

Thank you for your thoughtful and constructive comments. Please find our responses to each point below. We have addressed these in both the revised manuscript and our replies:

1. Heterogeneity over 50 years and evolving definitions:

We agree that including nearly five decades of research increases the risk of heterogeneity in terms of device maturity, diagnostic standards, and study designs. In our revised manuscript, we have expanded the discussion to explicitly acknowledge this issue **(Page 19 , line 2 - 7)**. We now note that:

- Earlier studies often lacked standardised criteria for glaucoma diagnosis (e.g. predating ISGEO),
- Device types have evolved significantly (from analogue tonometers to digital, AI-assisted platforms),
- The outcomes and reference standards used have varied across time.

We also highlight that more than half of included studies were published in the last decade, reinforcing the observation that the literature is expanding at a faster pace.

2. Reason why the pace of literature is increasing:

In the discussion **(Page 18 , line 4)**, we now reflect on possible reasons behind the acceleration of research in this field:

- **Technological innovation**: Advances in miniaturisation, convenience, wireless communication, and AI have enabled more sophisticated portable devices.
- **Patient empowerment and telemedicine trends**: There is growing interest in home-based monitoring and self-testing, especially among tech-literate populations and individuals with disability.
- **Healthcare system pressures**: Ageing populations and workforce shortages have prompted efforts to decentralise eye care and triage more effectively at the primary level.
- **The COVID-19 pandemic** further catalysed interest in remote diagnostics.

These factors combined are likely driving both research and industry efforts.

3. Study populations and target groups:

There are variety of target groups in the studies reviewed. However we agree that older, less mobile individuals and those in underserved settings will benefit more from these portable devices. In our revised discussion **(Page 17, line 36)**, we now reflect on:

- The median average age of participants was 55years.
- The urban and high-income country bias in study settings,
- The importance of more studies to evaluate these tools in nursing homes, home settings, or remote/rural populations.

We also argue that future research should prioritise these groups, who may benefit most from portable diagnostics.

4. Use in underdeveloped areas:

We have expanded the section on representativity and potential use in low-resource settings **(Page 17 , line 33).**

The revised manuscript now discusses:

The practical advantages of portable, battery-operated, low-maintenance devices in areas lacking fixed infrastructure seems promising. Coupled with its importance of co-developing workflows with community health workers and non-ophthalmic personnel. This underlines the need for future real-world implementation research to explore combined diagnostic pathways using multiple portable tools, especially in LMIC contexts, as proof-of-concept. Data alone may not reflect feasibility or scalability.

5. Agreement studies and the learning curve bias:

This is an important point. We now explicitly address this in our limitation (**Page 2, Line 30**) and discussion section **(Page 18, line 20):**

- Many agreement studies failed to mention participants prior exposure to the reference standard (e.g. Humphrey Field Analyser), potentially giving them an advantage compared to unfamiliar comparator devices.
- Learning curve of new or portable devices should be accounted for in the studies.
- As a result, reported agreement may not be accurate, particularly for perimetry measurements requiring users actions, feedback or alignment.

We suggest future studies should assess test–retest reliability across different levels of user experience.
